# Validity of the TBApp mobile application for self-care management for people with tuberculosis

**DOI:** 10.1590/0034-7167-2023-0195

**Published:** 2024-06-14

**Authors:** Lara Bezerra de Oliveira de Assis, Denise Maria Guerreiro Vieira da Silva, Jucimar Maia da Silva, Edinilza Ribeiro dos Santos, Jair dos Santos Pinheiro, Daniel Souza Sacramento, Hermann Jacques Hernani de Oliveira, Amélia Nunes Sicsú

**Affiliations:** I Universidade do Estado do Amazonas. Manaus, Amazonas, Brazil; II Secretaria Municipal de Saúde de Manaus. Manaus, Amazonas, Brazil; III Fundação de Vigilância em Saúde Dra. Rosemary Costa Pinto. Manaus, Amazonas, Brazil

**Keywords:** Mobile Applications, Tuberculosis, Primary Health Care, Validation Study, Self Care, Aplicaciones Móviles, Tuberculosis, Atención Primaria de Salud, Estudio de Validación, Autocuidado

## Abstract

**Objectives:**

to describe the validity process of the TBApp mobile application for self-care management for people with tuberculosis linked to Primary Health Care.

**Methods:**

methodological research developed with ten expert judges, carried out virtually. The application was assessed in relation to content and technology quality in seven domains (objectivity; structure and appearance; relevance; functionality; reliability; usability; and efficiency), using an instrument with a Likert scale.

**Results:**

TBApp was considered valid, relevant, functional, reliable and effective by expert judges. The objectives, structure and presentation and relevance domains presented an overall Content Validity Index of 0.93, and the functionality, reliability, usability and efficiency domains presented characteristics and sub-characteristics values greater than 0.80.

**Conclusions:**

TBApp is a creative and innovative tool that can be used by people with TB and disseminated in the scientific community.

## RESUMEN

**Objetivos:**

describir el proceso de validación de la aplicación móvil TBApp para la gestión del autocuidado de personas con tuberculosis, vinculada a la atención primaria de salud.

**Métodos:**

investigación metodológica desarrollada con diez jueces expertos, realizada de forma virtual. La aplicación fue evaluada en relación con la calidad del contenido y la tecnología en siete dominios (objetividad; estructura y apariencia; relevancia; funcionalidad; fiabilidad; usabilidad; y eficiencia), mediante un instrumento con escala Likert.

**Resultados:**

la TBApp fue considerada válida, relevante, funcional, confiable y eficaz por los jueces expertos. Los dominios de objetivos, estructura y presentación y relevancia presentaron un Índice de Validez de Contenido general de 0,93, y los dominios de funcionalidad, confiabilidad, usabilidad y eficiencia presentaron características y subcaracterísticas valores superiores a 0,80.

**Conclusiones:**

la TBApp es una herramienta creativa e innovadora que puede ser utilizada por personas con TB y difundida en la comunidad científica.

## INTRODUCTION

Tuberculosis (TB) remains a serious public health concern responsible for the majority of deaths attributable to a single infectious disease in the world, despite being a preventable and curable disease^(1, 2, 3)^. In 2021 alone, it is estimated that 10.6 million people became ill from TB worldwide. The incidence rate increased by 3.6% between 2020 and 2021, changing a previous trend of falling by approximately 2% per year^(3)^. In Brazil, in 2022, 78,057 new cases of TB were registered, with an incidence rate of 36.3 cases per 100 thousand inhabitants and an increasing number of deaths from this disease^(4)^.

Lack of adherence to medication is a major obstacle to the elimination of TB, resulting in prolonged infectiousness, relapse, emergence of multidrug resistance and increased risk of treatment inadequacy^(5, 6, 7, 8)^. In Brazil, in 2021, 14% of people with TB interrupted treatment and there were around 5% of records with unknown information regarding the outcome^(4)^.

The main strategy for improving adherence to TB treatment in Brazil is Directly Observed Treatment (DOT), however this strategy has limitations^(9, 10)^. DOT requires healthcare professionals to regularly travel to the home of people with TB or travel to the health service, resulting in financial and time costs and, for some people, loss of autonomy and privacy^(11)^. This scenario shows the need to include new strategies that improve monitoring of sick people and their adherence to treatment.

The End TB Strategy, approved by the World Health Assembly in 2014 and by Brazil in 2017, states that integrated, people-centered prevention and care, bold policies and support systems for those affected by TB, together with innovation and research intensification, are imperative to significantly reduce the disease incidence and mortality^(3, 12)^.

New methods of promoting treatment adherence, especially electronic ones, have been developed and considered promising, constituting efforts to develop a solid base of encouragement and support for adherence to TB treatment^(13, 14, 15)^. In a technological prospecting study, carried out in 2022, to map mobile applications on TB prevention, diagnosis and treatment, 42 applications were found around the world, the majority aimed at the general public or medical professionals. This number points to a growing use of these tools in the care of people with TB, but with room for new applications^(16)^. A scoping review, carried out by researchers from India with the aim of exploring mobile health (mHealth) technologies available for TB management in India, concluded that there is a preponderance of applications focused on TB treatment and medication monitoring and that there is a shortage of applications for educating the public about preventing TB^(17)^.

Researchers’ experiences in different areas of action against TB in the state of Amazonas, Brazil, generated interest and concerns and drove the search for solutions to improve adherence to treatment among people undergoing treatment for TB, seeking to contribute to the End TB Strategy, which has as its pillars technological innovation as a strategy to eradicate the disease^(3)^.

From this perspective, a mobile application was developed for self-care for people with pulmonary TB followed in Primary Health Care (PHC) called TBApp^(18)^. The application was developed in Manaus, Amazonas, in the Graduate Program in Public Health

Nursing (ProEnSP) with support from the *Universidade do Estado do Amazonas* Laboratory of Technology, Innovation and Creative Economy (UEA/LUDUS). Its development included the collaboration of different members of the Tuberculosis Control Program Center of Manaus, Amazonas, with the intention of making the application appropriate to the reality of TB in the Amazon, since Manaus is among the capitals with the highest number of cases of TB in Brazil^(19)^.

TBApp is considered a technology for managing self-care and case management in TB treatment by the healthcare team, with daily audio message features, such as reminders to take medication, motivating messages, space with educational questions about the disease, in addition to monitoring by management in real time. This will be possible as users registered on the application will be monitored by the healthcare team, who will be able to intervene whenever necessary.

Users, when registering on TBApp, will inform the scheduled time to take daily doses and the weight to adjust the number of tablets, in accordance with national recommendations that will be validated by the healthcare team. Upon registration, users will receive a reminder message at the scheduled time “Have you taken your medication today?”, with the “Yes” or “No” answer option, directing to another screen to select pills and confirm. If it does not register correctly, the application will send an alert message for a period of up to 12 hours, not allowing retroactive registrations after this period, and, if registration is carried out correctly, users will receive a motivating and informative message, such as “Continue treatment! People who live with you need to be checked!”. The absence of records or incomplete records will be monitored through a web interface by the service to carry out active search and monitoring.

TBApp was developed for Android and iOS mobile devices in JavaScript language, and can be used offline and/or when there is a signal of internet connectivity, and the data is sent to the cloud. To incorporate TBApp into health services, it was considered essential to be submitted to a validity process by experts in the field so that it can be a reliable technology and achieves the objectives for which it was designed.

## OBJECTIVES

To describe the content and quality validity process of the TBApp mobile application for managing self-care for people with TB linked to PHC.

## METHODS

### Ethical aspects

The research was approved by the UEA Research Ethics Committee, and all requirements for the protection of participants in scientific research involving human beings were met.

### Study design, period and place

This is methodological research developed with a focus on the TBApp content and quality validation process. The mobile application was created based on technological development research in four stages (conception, elaboration, construction and transition), using the Rational Unified Process (RUP) method^(20)^.

Digital technology production requires that it undergo an objective, transparent and standards-based assessment of digital health products, subject to validation of its content by experts in the field^(21)^. The tool validation stage took place virtually from February to July 2021. The method was guided by using the Equator network Standards for QUality Improvement Reporting Excellence (SQUIRE 2.0)^(22)^.

### Population and selection criteria

The application assessment included the participation of ten expert judges: five coordinators of the Tuberculosis Control Program (TCP) and five researchers in the area of TB, two from each region of Brazil (one researcher and one TCP coordinator). The inclusion of judges from all regions of Brazil was intended to verify whether it would be valid for primary care in the country. The criteria for assessing software from the Brazilian Association of Technical Standards (ABNT - *Associação Brasileira de Norma Técnica*) ISO/IEC 25062:2011 were used as a parameter for the number of experts, which recommends at least eight participants^(23)^.

To form the group of research experts, they had to have a doctorate and at least three years of experience in the area of TB research. The choice of these research judges was initially carried out in the Directory of Research Groups in Brazil of the Brazilian National Council for Scientific and Technological Development (CNPq - *Conselho Nacional de Desenvolvimento Científico e Tecnológico*) with the search “tuberculosis”, applying search on group name and search line name. The groups were identified by region and then the *Curriculum Lattes* of the researchers from the research groups were analyzed to assess the established criteria. A total of 28 judges were selected and sent an email inviting them to participate in the study. Of these, 17 accepted and signed the Informed Consent Form (ICF) online, however, after sending the link and assessment instrument, only five judges responded to the study.

As for TCP coordinators, they should have at least specialization in the area and two years of experience in TB management. The judges participating in the sample were selected by convenience, through network or chain sampling. The inclusion of these judges was carried out based on the indication of the TB coordinator in the city of Manaus, Amazonas, by providing the email address of the TB coordinators in each Brazilian state, covering the five regions. An invitation to participate was sent to all nominated coordinators. When one of these guests responded that they could not participate in the study, they were asked to nominate another participant from the same region who met the study criteria. This process was repeated until the five previously established judges were obtained.

### Study protocol

The validation process by expert judges in the area was carried out using a structured instrument in Google Forms, consisting of two parts. The first part included data on participant characteristics, such as age, sex, profession, time since training, time working in the area, area of qualification. The second part was composed of seven domains, three related to content: objectives (with five questions); structure and presentation (with seven questions); and relevance (with five questions); and four domains related to technology: functionality (with three questions); reliability (with two questions); usability (with three questions); and efficiency (with two questions), totaling 27 questions.

The content-related domains correspond to the instrument adapted by Oliveira^(24)^, and those related to technology correspond to the software validation instrument adapted by Tibes-Cherman^(25)^. A Likert-type scale was used for the answers, with the respective options: totally disagree (TD); partially disagree (PD); partially agree (PA); totally agree (TA). At the end of each domain, a space was inserted so that judges could give their suggestions for improving the application.

An email with an invitation letter was sent to judges with the ICF. If accepted, a link to the application and assessment instrument was sent for handling, with a ten-day deadline for return.

### Analysis of results, and statistics

Judges’ assessments were entered into a Microsoft Office Excel spreadsheet for statistical analysis. Relative frequency and the Content Validity Index (CVI) were used for domains related to content^(26)^. The CVI was used to assess the degree of agreement among judges. The CVI of each item was calculated separately using the following formula for calculation: CVI = ∑ PA *and* TA *answers* ⁄ ∑ ⬚ *totalanswers* (*TD* + *PD* + *PA* + *TA*). To calculate the CVI of each domain, the CVI of all items in the domain was added and divided by the number of items in that domain. From the sum of the indexes obtained from each domain divided by the number of domains, the general CVI was obtained. For the present study, a CVI greater than 0.7 was considered valid.

To assess the domains related to the technical quality of TBApp, we used the calculation of the values of each characteristic and respective subcharacteristic, in accordance with the rule proposed by ABNT NBR ISSO/IEC 14598-6^(27)^, using the formula:

$$\begin{array}{c} \begin{array}{c} CV=\sum V_{sc/nsc}\\ SCV=\sum m/\left(n-nd\right) \end{array} \end{array}$$

In the formula, CV is the measured characteristic values; SCV is the measured subcharacteristic values; nsc is the subcharacteristic number; m (1) if the answer is positive and (0) if the answer is negative; n is the total number of measurements; nd is the number of discarded questions. To transform CV and SCV values into indices, their values were multiplied by 100.

The answers totally agree and partially agree were considered as positive answers, and partially disagree or totally disagree as negative answers^(28)^. Expected values for characteristics and subcharacteristics were considered, values above 70%, according to ABNT NBR ISSO/IEC 14598-6^(27)^.

Judges’ suggestions were categorized into positive and negative points. The judges were coded with the letter J and numbered from 1 to 10, in order to maintain anonymity. Subsequently, suggestions for signaling whether they would be accepted or not were summarized.

## RESULTS

The ten TBApp evaluators were nurses, two from each region of Brazil, nine of whom were women, with a mean age of 44.5 years, ranging between 31 and 70 years. Six judges had between 10-20 years of training and four had more than 20 years of training. Regarding degrees, five were doctors, three were master’s holders and two were experts, with a predominant area of specialization in public health. Taking as a reference participant origin in relation to the five regions of Brazil, there was no difference between the answers of members from each region.

Table 1 shows the distribution of content assessment made by expert judges regarding objective, structure, presentation and relevance. All domains presented CVI above 0.9, i.e., not requiring additional adjustment. The “The application is appropriate to the proposed target audience’s sociocultural level” item from the “structure” domain obtained agreement equal to 70% and CVI equal to 0.70, being considered valid; however, considering that it was the item that obtained the CVI lower, adjustments were made, as suggested by expert judges.

**Table 1:** TBApp content assessment by expert judges regarding objectivity, structure and presentation, relevance, Manaus, Amazonas, Brazil, 2021

Assessed domains	Item	TD	PD	PA	TA	CVI	Overall CVI
Objective	It is consistent with the needs of people sick with TB	0	0	30%	70%	1	0.96
	It is coherent from the point of view of the process of combating TB	0	0	50%	50%	1	
	Promotes change in behavior and attitude	0	10%	60%	30%	0.9	
	It can be disseminated in scientific circles in the area of TB	0	0	0%	100%	1	
	It meets the objectives of institutions that work with TB	0	10%	30%	60%	0.9	
Structure and presentation	The application is adequate for people sick with TB	0	0	50%	50%	1	0.91
	Messages are presented clearly and objectively	0	0	50%	50%	1	
	The information presented is scientifically correct	0	0	30%	70%	1	
	The application is appropriate to the proposed target audience’s sociocultural level	0	30%	30%	40%	0.7	
	The application content has a logical sequence	0	10%	10%	80%	0.9	
	The information is well structured in terms of agreement and spelling	0	10%	60%	30%	0.9	
	The writing style corresponds to the target audience’s knowledge level	0	10%	50%	40%	0.9	
Relevance	Frequently asked questions portray key aspects that should be reinforced	0	0	30%	70%	1	0.92
	The application allows learning about the disease	0	10%	40%	50%	0.9	
	The application encourages people suffering from TB to acquire knowledge for their own care	0	10%	30%	60%	0.9	
	The application addresses the necessary topics for people suffering from TB	0	10%	30%	60%	0.9	
	It is adequate for use by people sick with TB	0	10%	50%	40%	0.9	
	The resources available in the application are adequate	0	0	40%	60%	0.9	
OVERALL CVI: 0.96 (Domain 1) + 0.91 (Domain 2) + 0.92 (Domain 3) / 3 = 0.93							

*TD - totally disagree; PD - partially disagree; PA - partially agree; TA - totally agree; CVI - Content Validity Index; TB - tuberculosis.*

Table 2 shows the distribution of the TBApp assessment carried out by expert judges in relation to technology, including the functionality, reliability, usability and efficiency domains. All characteristics and subcharacteristics presented values equal to or greater than 0.80, i.e., in all responses, a positive assessment was obtained. The best assessed domain was functionality, and with lower SCV and CV was the usability domain, but still with satisfactory SCV and CV, indicating that the final version of the application was considered suitable for use.

**Table 2:** Quality assessment of TBApp software by judges regarding functionality, reliability, usability and efficiency, Manaus, Amazonas, Brazil, 2021

Assessed domains	Item	Answered received		Final value	
		Positive	Negative	SCV	CV
Functionality	The application has the main functions to assist with adherence to TB treatment	9	1	90%	90%
	The application is accurate in performing its functions	8	2	80%	
	The application has access security through passwords	10	0	100%	
Reliability	The application displays messages when failures occur	9	1	90%	85%
	The application informs the user of invalid data entry	8	2	80%	
Usability	It is easy to understand the concept and application of the software	9	1	90%	83%
	It is easy to learn how to use the app	8	2	80%	
	The app offers help clearly	8	2	80%	
Efficiency	Software runtime is adequate	8	2	80%	90%
	The resources available in the application are adequate	10	0	100%	

*SCV - measured subcharacteristic values; CV - measured characteristic values.*

In Chart 1, examples of changes made to TBApp following suggestions from evaluators are presented. Most suggestions were related to the structure and presentation domains and the “The application is appropriate to the proposed target audience’s sociocultural level” item, which obtained CVI = 0.70.

**Chart 1:** Examples of changes made to the application’s sub-items according to suggestions from the judges, Manaus, Amazonas, Brazil, 2021

Item	Subitems	
	Previous version	Final version
Who are the groups most susceptible to developing tuberculosis?	People living with HIV/AIDS; Persons deprived of liberty; Homeless population; Indigenous; Healthcare professionals; Contacts of tuberculosis patients, mainly children and older adults, and people with diabetes.	People with HIV/AIDS; Inmates; Homeless people; Indigenous; Healthcare professionals; People who live with tuberculosis patients, especially children and the elderly, and people with diabetes.
I started taking the medication, but I felt bad. What should I do?	CONTINUE TO TAKE YOUR MEDICATIONS. It is important that you return to work immediately so that healthcare professionals can assess you.	Return to the healthcare facility immediately so that a healthcare professional can assess you. Do not stop treatment.
Do I need to stop drinking alcoholic beverages during tuberculosis treatment?	YES. You cannot drink alcoholic beverages, such as cachaças, beers, whiskey, wines, and others, during treatment, as there is a risk of complications, such as hepatitis. However, there are patients who are unable to stop drinking, in this case, it is recommended to inform the healthcare professional who is monitoring you and be sure to take your medications.	It is advisable yes. You cannot drink alcoholic beverages, such as cachaças, beers, whiskey, wines, and others, during treatment, as there is a risk of complications, such as hepatitis. However, if you have difficulty stopping drinking or stopping using other drugs, talk to your healthcare professional.
Why is it important to take an HIV test?	Tuberculosis is an infectious disease that is predominant in people living with HIV, due to the compromised immunity of these patients, and is also the main cause of death in this group. Therefore, it is important that you undergo an HIV test due to the relationship between the two diseases.	HIV is a silent disease that compromises the body’s defense against other diseases, such as tuberculosis. When the patient has both diseases (TB and HIV), the chance of worsening is greater. Therefore, early detection through HIV testing is important.
Why do people who live with me have to be tested?	Because the family are generally the people who live with you the most, therefore, they are the ones who are most at risk of becoming infected and falling ill, as they maintained contact before the diagnosis and the start of treatment. It is important that everyone is tested, even if they do not have symptoms, as there is a possibility that their contacts are infected and at risk of developing the disease in the coming years.	Because people who live with you (family) are in the same environment, they are at greater risk of becoming infected and falling ill, because before diagnosis and treatment begins, you release the bacteria that cause the disease into the environment. In this case, it is important that everyone is examined, those with symptoms through sputum examination, and those who do not present symptoms of tuberculosis through the Tuberculin Skin Test (TST) and chest x-ray, as there is a possibility that their contacts are infected and at risk of developing the disease in the coming years.
I am undergoing tuberculosis treatment, what precautions should I take in relation to COVID-19? (question about COVID-19 inserted)	Inserted in the second version.	It is important to maintain treatment and take the following precautions: wearing masks, washing hands with soap and water, using alcohol gel, avoiding crowds, and checking your vaccination schedule against COVID-19. In cases of symptoms such as fever, dry cough, tiredness, sore throat, runny nose, lack of smell and taste, seek the service.

In Chart 2, the positive and negative points of the application are presented according to expert judges.

**Chart 2:** Positive and negative points highlighted by expert judges, Manaus, Amazonas, Brazil, 2021

Domains	Positive points	Negative points
1. Objective	Assistance for institutions and healthcare professionals in dealing with TB and difficulties in adhering to treatment.	It accepts the recording of medication intakes, but does not guarantee observation of medication intake.
2. Structure and appearance	Clear and objective texts.	Very technical answers considering the profile of people affected by TB.
3. Relevance	I would recommend it to people who are or are treating people with TB.	Not highlighted.
4. Functionality	The application is an excellent tool to be used during treatment.	Not highlighted.
5. Reliability	Correct information.	Not highlighted.
6. Usability	Using the application is simple, easy to use, with objective and clear information.	The most vulnerable population may not have access to or find it easy to use the tool.
7. Efficiency	Good response time and adequate resources available.	Difficulty recovering the password.

*TB – tuberculosis.*

## DISCUSSION

Mobile applications created based on scientific research are relevant for implementation in practice, as they tend to be analyzed and tested by professionals who know the target audience’s needs^(29, 30)^. The process of validating a technology, when produced appropriately and validated, may contribute to modifying the reality of the subjects for whom it is intended^(31)^.

TBApp was validated against seven domains, organized into content and technology. The results of this validation allow us to affirm that TBApp, from the point of view of researchers in the area and those who are part of the management of care practices for people with TB, linked to TCP, has adequate content and the technology meets the necessary characteristics for an application. The literature points out that the evaluative and suggestive process based on different views by judges tends to contribute to the technology’s technical-scientific scope^(32, 33)^.

Another relevant aspect of the validation carried out is that TBApp was assessed by nurse judges from the five regions of Brazil with experience in the area of public health and TB, and there was no difference between their answers, indicating that it could be suitable for use throughout the country.

Recognizing the quality of the application validated by judges had as its main suggestion the change in the language used, since some evaluators considered that it was not always clear to the target audience, which explains the assessment given to the question “Is the application appropriate to the proposed target audience’s sociocultural level”, which obtained the lowest CVI (0.7). The suggestions were considered relevant by the researchers who made changes to the final content of TBApp.

Language was also highlighted as a relevant adjustment item in a mobile application validation study to monitor the treatment of latent TB infection, whose changes considered patients’ reports related to clarity and simplicity^(34)^, showing the importance of digital technologies having clear and accessible language, ensuring understanding by anyone, regardless of socio-cultural-educational level.

Many positive points were highlighted by the evaluators in relation to the tool, being unanimous about the dissemination of the tool in the scientific community. Despite the growth of applications in the health sector, the vast majority are created by private developers, are not linked to research projects and are not publicized in scientific circles^(25)^.

One of the evaluators highlighted that TBApp does not guarantee that medication is being taken. It is worth highlighting that the proposed technology is not intended to replace DOT, observation of medication intake must be carried out by a healthcare professional or a trained person at least three times a week in the attack phase and at least twice in the maintenance phase, as recommended by the World Health Organization and Ministry of Health guidelines^(9)^. TBApp’s proposal is to be a self-care tool capable of providing empowerment to users during their treatment, helping them with reminders and encouragement and with more information about their disease and the importance of regular treatment.

Counseling and reminder methods have been considered suitable for people with active TB, highlighting digital adherence technologies^(6)^. Furthermore, systematic review results showed that there are higher treatment success rates with the use of adherence interventions such as education, reminders and digital health technologies, which reinforces the importance of the TBApp developed, as it brings reminders to take medication and motivating messages as well as educational content about the disease, tests and treatment^(35)^.

It is extremely important to create mobile technologies in nursing, such as the one proposed in this study; however, these technologies are not intended to replace nursing consultations, but they must act in a complementary way to consultations, being a strategy that enables patient empowerment over their health condition and their role in improving their quality of life^(36)^.

Technologies that innovate communication with people served in the healthcare sector are expanding access to information and enhancing therapeutic adherence^(37)^. TBApp was considered a suitable application for use in PHC. However, it is important to consider existing social and economic barriers, because, although the majority of the Brazilian population has cell phones, not everyone has the availability of smartphones or other mobile devices or even devices with sufficient technology to allow downloading applications^(38)^. An alternative suggested by researchers was the provision of smartphones to people suffering from TB during treatment associated with training on how to use the application, with the possible return of the device after successful completion of treatment^(38, 39)^. This suggestion, although interesting, does not express the reality of Brazilian municipalities, which are far from the possibility of having budgets that allow these acquisitions.

The development of TBApp is aligned with the United Nations Sustainable Development Goals for 2030, in particular its Objective 3.3: by 2030, to end the epidemics of AIDS, TB, malaria and neglected tropical diseases and combat hepatitis, water-borne diseases and other communicable diseases^(40)^.

TBApp’s contribution is to offer an easily accessible device to people with TB that will help them better control their health condition and provide healthcare professionals, especially nurses, with a tool to obtain information and help people with TB, even remotely, interacting through the application. Therefore, it is considered that the validated application can be used in PHC, contributing to the provision of pertinent and quality information.

The validated technology differs from others developed in Brazil and combines different functionalities of technologies produced in other countries^(16, 17)^. The developed application, TBApp, is considered a creative and innovative tool for people sick with TB in PHC in the city of Manaus. The application allows daily recording of medication taken by sick people, in addition to reminder messages, with an educational interface on the general aspects of TB and an interface for the Web system (TBSite) for the restricted use of healthcare professionals and managers and monitoring of cases monitored by TBApp, allowing the generation of statistics by unit, district and municipality.

### Study limitations

As a limitation of this study, it is possible to indicate the non-participation of healthcare professionals who care for people with TB, which could contribute to an operational analysis in relation to the use of technology. However, it is important to highlight that this issue will be considered in future studies, seeking the inclusion of these professionals to enrich the understanding of the application implementation and impact on the practice of professionals who work directly in caring for people with TB.

### Contributions to nursing

The results of validating TBApp for self-care management for people with TB show that it has the potential to improve the care provided by nurses in PHC. The application in question represents an additional alternative in care centered on people with TB, since its applicability was previously identified. The dissemination and adoption of technology by the target population can promote self-care, increase knowledge on the topic and bring significant improvements in quality of life.

## CONCLUSIONS

This study assessed TBApp for self-care for people with TB in PHC. The application, according to judges’ assessment, proved to be effective, functional, reliable, with good usability and relevance.

The development of this proposal has the potential to impact the disease morbidity and mortality by promoting self-care among people sick with TB as well as being a case monitoring tool, helping control programs achieve operational indicators to eliminate the disease as a public health problem.

The participation of experts involved in management and research on TB enhanced application quality. It is hoped that this technology can serve as a reference and be used in other locations in Brazil, as well as in countries with a high incidence of TB, due to the importance of digital health tools.

Further research to assess the tool with the target audience based on its use in services may identify the impact of TBApp on disease indicators, in addition to mobilizing the development of new technologies for TB control.

## Data Availability

https://doi.org/10.48331/scielodata.TCLER5
