# Supplementary material for: Validity of the TBApp mobile application for self-care management for people with tuberculosis
Source: Rev Bras Enferm. 2024 Jun 14;77(2):e20230195. doi: 10.1590/0034-7167-2023-0195 (PMC11178314; doi:10.1590/0034-7167-2023-0195)
Supplement: Translation

# Translation: Validação do aplicativo móvel TB*App* para a gestão do autocuidado de pessoas com tuberculose

**DOI:** 10.1590/0034-7167-2023-0195pt

**Authors:** Lara Bezerra de Oliveira de Assis, Denise Maria Guerreiro Vieira da Silva, Jucimar Maia da Silva, Edinilza Ribeiro dos Santos, Jair dos Santos Pinheiro, Daniel Souza Sacramento, Hermann Jacques Hernani de Oliveira, Amélia Nunes Sicsú

**Affiliations:** I Universidade do Estado do Amazonas. Manaus, Amazonas, Brasil; II Secretaria Municipal de Saúde de Manaus. Manaus, Amazonas, Brasil; III Fundação de Vigilância em Saúde Dra. Rosemary Costa Pinto. Manaus, Amazonas, Brasil

**Keywords:** Aplicativos Móveis, Tuberculose, Atenção Primária à Saúde, Estudo de Validação, Autocuidado

## Abstract

**Objetivos:**

descrever o processo de validação do aplicativo móvel TB*App* para a gestão do autocuidado de pessoas com tuberculose vinculadas à Atenção Primária à Saúde.

**Métodos:**

pesquisa metodológica, desenvolvida com dez juízes especialistas, realizada por meio virtual. O aplicativo foi avaliado em relação a conteúdo e qualidade da tecnologia, em sete domínios (objetividade; estrutura e aparência; relevância; funcionalidade; confiabilidade; usabilidade; e eficiência), por meio de instrumento com escala tipo Likert.

**Resultados:**

o TB*App* foi considerado válido, relevante, funcional, confiável e eficaz pelos juízes especialistas. Os domínios de objetivos, estrutura e apresentação e relevância apresentaram Índice de Validade de Conteúdo global de 0,93, e os domínios de funcionalidade, confiabilidade, usabilidade e eficiência apresentaram valores de características e subcaracterísticas maiores que 0,80.

**Conclusões:**

o TB*App* é uma ferramenta criativa e inovadora, podendo ser utilizado por pessoas com TB e divulgado no meio científico.

## INTRODUÇÃO

A tuberculose (TB) continua como um grave problema de saúde pública responsável pela maioria das mortes atribuíveis a uma única doença infecciosa no mundo, apesar de ser uma doença evitável e curável^(1, 2, 3)^. Somente em 2021, estima-se que 10,6 milhões de pessoas adoeceram por TB no mundo. A taxa de incidência aumentou 3,6% entre 2020 e 2021, mudando uma tendência anterior de queda de aproximadamente 2% ao ano^(3)^. No Brasil, em 2022, foram registrados 78.057 casos novos de TB, com um coeficiente de incidência de 36,3 casos por 100 mil habitantes e crescente número de mortes por essa doença^(4)^.

A falta de adesão à medicação se configura um grande obstáculo para a eliminação da TB, resultando em infecciosidade prolongada, recidiva, surgimento de multirresistência e aumento do risco de inadequabilidade do tratamento^(5, 6, 7, 8)^. No Brasil, em 2021, 14% das pessoas com TB interromperam o tratamento e houve cerca de 5% dos registros com informação ignorada quanto ao desfecho^(4)^.

A principal estratégia para melhoria da adesão ao tratamento da TB no Brasil é o Tratamento Diretamente Observado (TDO), porém tal estratégia apresenta limitações^(9, 10)^. O TDO requer o deslocamento regular do profissional de saúde à residência da pessoa com TB ou seu deslocamento ao serviço de saúde, resultando em gastos financeiros e de tempo e, para algumas pessoas, perda da autonomia e privacidade^(11)^. Esse cenário mostra a necessidade de inclusão de novas estratégias que melhorem o acompanhamento à pessoa adoecida e a sua adesão ao tratamento.

A Estratégia *End TB*, aprovada pela Assembleia Mundial da Saúde, em 2014, e pelo Brasil, em 2017, afirma que a prevenção e os cuidados integrados e centrados na pessoa, políticas ousadas e sistemas de apoio e suporte aos afetados pela TB, juntamente com a intensificação da inovação e da pesquisa, são imperativos para reduzir significativamente a incidência e mortalidade da doença^(3, 12)^.

Novos métodos de promoção da adesão ao tratamento, especialmente eletrônicos, têm sido desenvolvidos e considerados promissores, constituindo esforços para desenvolver uma base sólida de estímulo e suporte para a adesão ao tratamento da TB^(13, 14, 15)^. Em estudo de prospecção tecnológica, realizado em 2022, para mapear os aplicativos móveis sobre prevenção, diagnóstico e tratamento da TB, foram encontrados 42 aplicativos em todo o mundo, a maioria destinada ao público em geral ou a profissionais da medicina. Esse número aponta um crescente uso dessas ferramentas no cuidado às pessoas com TB, mas com espaço para novos aplicativos^(16)^. *Scoping review*, realizada por pesquisadores da Índia com o objetivo de explorar as tecnologias de saúde móvel (*mHealth*) disponíveis para o manejo da TB na Índia, concluiu que há uma preponderância de aplicativos centrados no tratamento da TB e na monitorização de medicamentos e que existe escassez de aplicativos para a educação do público no sentido de prevenir a TB^(17)^.

Experiências das pesquisadoras em diferentes âmbitos de ação contra a TB no estado do Amazonas, Brasil, geraram interesse e inquietações e impulsionaram a busca por soluções para melhorar a adesão ao tratamento das pessoas em tratamento para a TB, buscando contribuir para a Estratégia *End TB*, que tem como pilares a inovação tecnológica como estratégia para erradicação da doença^(3)^.

Nessa perspectiva, foi desenvolvido um aplicativo móvel para o autocuidado de pessoas com TB pulmonar acompanhadas na Atenção Primária à Saúde (APS) denominado TB*App* ^(18)^. O aplicativo foi desenvolvido em Manaus, Amazonas, no Programa de Pós-Graduação em Enfermagem em Saúde Pública (ProEnSP) com apoio do Laboratório de Tecnologia, Inovação e Economia Criativa da Universidade do Estado do Amazonas (LUDUS/UEA). Seu desenvolvimento contou com a colaboração de diferentes integrantes do Núcleo do Programa de Controle de Tuberculose de Manaus, Amazonas, com a intenção de tornar o aplicativo adequado à realidade da TB na Amazônia, uma vez que Manaus está entre as capitais com maior número de casos de TB no Brasil^(19)^.

O TB*App* é considerado uma tecnologia de gestão do auto-cuidado e gestão de casos em tratamento de TB pela equipe de saúde, com funcionalidades de mensagens sonoras diárias, como lembretes da tomada da medicação, mensagens motivadoras, espaço com questões educativas sobre a doença, além do monitoramento pela gestão em tempo real. Isso será possível, uma vez que os usuários cadastrados no aplicativo serão acompanhados pela equipe de saúde, que poderá intervir sempre que necessário.

O usuário, ao realizar o cadastro no TB*App*, informará o horário programado para tomar as doses diariamente e o peso para ajuste na quantidade de comprimidos, conforme as recomendações nacionais que serão validadas pela equipe de saúde. A partir do cadastro, o usuário receberá mensagem lembrete no horário programado “Já tomou seus remédios hoje?”, com opção de resposta “Sim” ou “Não”, direcionando para outra tela para seleção das pílulas e confirmação. Caso ele não registre corretamente, o aplicativo enviará mensagem de alerta por um período de até 12 horas, não permitindo registros retroativos após esse prazo, assim como, caso seja realizado o registro corretamente, o usuário receberá mensagem motivadora e informativa, como “Continue o tratamento! Pessoas que moram com você precisam ser examinadas!”. A ausência de registros ou registros incompletos será monitorada através de uma *interface web* pelo serviço para realização de busca ativa e acompanhamento.

O TB*App* foi desenvolvido para dispositivos móveis Android e iOS na linguagem *JavaScript,* podendo ser utilizado *offline* e/ou quando houver sinal de conectividade de internet, e os dados são enviados para a nuvem. Para incorporação do TB*App* nos serviços de saúde, foi considerado imprescindível ser submetido a um processo de validação por especialistas da área, de modo que possa ser uma tecnologia confiável e atinja os objetivos para o qual foi projetado.

## OBJETIVOS

Descrever o processo de validação de conteúdo e de qualidade do aplicativo móvel TB*App* para a gestão do autocuidado de pessoas com TB vinculadas à APS.

## MÉTODOS

### Aspectos éticos

A pesquisa foi aprovada pelo Comitê de Ética em Pesquisa da UEA, e cumpriram-se todas as exigências para a proteção dos participantes de pesquisa científica que envolvem seres humanos.

### Desenho, período e local do estudo

Trata-se de pesquisa metodológica desenvolvida com foco no processo de validação de conteúdo e de qualidade do TB*App*. O aplicativo móvel foi criado a partir de uma pesquisa de desenvolvimento tecnológico em quatro etapas (concepção, elaboração, construção e transição), utilizando o método *Rational Unified Process* (RUP)^(20)^.

A produção de uma tecnologia digital requer que ela passe por uma avaliação objetiva, transparente e baseada em padrões de produtos digitais de saúde, sendo submetida a uma validação de seu conteúdo por *experts* da área^(21)^. A etapa de validação da ferramenta ocorreu de forma virtual no período de fevereiro a julho de 2021. O método foi orientado pelo uso do instrumento *Standards for QUality Improvement Reporting Excellence* (SQUIRE 2.0) da rede Equator^(22)^.

### População e critérios de seleção

A avaliação do aplicativo contou com a participação de dez juízes especialistas: cinco coordenadores do Programa de Controle de Tuberculose (PCT) e cinco pesquisadores da área da TB, sendo dois de cada região do Brasil (um pesquisador e um coordenador do PCT). A inclusão de juízes de todas as regiões do Brasil teve a intenção de verificar se ele seria válido para a atenção básica do país. Utilizou-se como parâmetro para o número de especialistas o critério para avaliação de *software* da Associação Brasileira de Norma Técnica (ABNT) ISO/IEC 25062:2011, que recomenda, no mínimo, oito participantes^(23)^.

Para compor o grupo de especialistas pesquisadores, esses tinham que possuir doutorado e, no mínimo, três anos de experiência na área de pesquisa em TB. A escolha desses juízes pesquisadores foi realizada inicialmente no Diretório dos Grupos de Pesquisa no Brasil do Conselho Nacional de Desenvolvimento Científico e Tecnológico (CNPq) com a busca “tuberculose”, aplicando busca em nome do grupo e nome da linha de pesquisa. Foram identificados os grupos por região e, em seguida, efetuada análise do *Curriculum Lattes* dos pesquisadores dos grupos de pesquisa para avaliação dos critérios estabelecidos. Foram selecionados 28 juízes, aos quais foi enviado e-mail com convite para participar do estudo. Desses, 17 aceitaram e assinaram o Termo de Consentimento Livre e Esclarecido (TCLE) *online*, no entanto, após o envio do *link* e instrumento de avaliação, somente cinco juízes responderam ao estudo.

Quanto aos coordenadores do PCT, esses deveriam possuir no mínimo especialização na área e dois anos de experiência na gestão da TB. Os juízes participantes da amostra foram selecionados por conveniência, por meio de uma amostragem em rede ou em cadeia. A inclusão desses juízes foi realizada a partir da indicação do coordenador de TB do município de Manaus, Amazonas, por meio do fornecimento do e-mail dos coordenadores de TB de cada estado brasileiro, contemplando as cinco regiões. Foi enviado convite para participar a todos os coordenadores indicados. Quando um desses convidados respondia que não poderia participar do estudo, era solicitado que indicasse outro participante da mesma região que atendesse aos critérios do estudo. Esse processo foi repetido até obter os cinco juízes previamente estabelecido.

### Protocolo do estudo

O processo de validação por juízes especialistas da área foi efetuado utilizando um instrumento estruturado no *Google Forms*, composto por duas partes. A primeira parte contemplava dados sobre a caracterização dos participantes, como idade, sexo, profissão, tempo de formação, tempo de atuação na área, área da titulação. A segunda parte foi composta por sete domínios, três relacionados ao conteúdo: objetivos (com cinco questões); estrutura e apresentação (com sete questões); e relevância (com cinco questões); e quatro domínios relacionados à tecnologia: funcionalidade (com três questões); confiabilidade (com duas questões); usabilidade (com três questões); e eficiência (com duas questões), totalizando 27 questões.

Os domínios relacionados ao conteúdo correspondem ao instrumento adaptado por Oliveira^(24)^, e os relacionados à tecnologia, ao instrumento de validação de *software* adaptado de Tibes-Cherman^(25)^. Foi utilizada como resposta uma escala tipo Likert, com as respectivas opções: discordo totalmente (DT); discordo parcialmente (DP); concordo parcialmente (CP); concordo totalmente (CT). No final de cada domínio, foi inserido um espaço para que os juízes pudessem dar suas sugestões de melhoria do aplicativo.

Foi enviado e-mail com carta convite aos juízes com o TCLE. Em caso de aceite, era enviado o *link* do aplicativo, para manuseio, e do instrumento de avaliação, sendo estipulado um prazo de dez dias para a devolução.

### Análise dos resultados e estatística

As avaliações dos juízes foram inseridas em planilha eletrônica no *Microsoft Office Excel* para análise estatística. Foram utilizados a frequência relativa e o Índice de Validade de Conteúdo (IVC) para os domínios relacionados ao conteúdo^(26)^. O IVC foi utilizado para avaliar o grau de concordância entre os juízes. Calculou-se o IVC de cada item separadamente utilizando para o cálculo a seguinte fórmula: IVC = ∑ *respostas* CP *e* CT ⁄ ∑ ⬚ *totaldasrespostas* (*DT* + *DP* + *CP* + *CT*). Pa r a o cálculo do IVC de cada domínio, somou-se o IVC de todos os itens do domínio e dividiu-se pelo número de itens do referido domínio. Da somatória dos índices obtidos de cada domínio divididos pelo número de domínios, obteve-se o IVC geral. Para o presente estudo, considerou-se válido IVC maior que 0,7.

Para avaliação dos domínios relacionados à qualidade técnica do TB*App*, utilizou-se o cálculo dos valores de cada característica e respectiva subcaracterística, de acordo com regra proposta pela ABNT NBR ISSO/IEC 14598-6^(27)^, usando a fórmula:


$$\begin{array}{c} \begin{array}{c} VC=\sum V_{sc/nsc}\\ VSC=\sum m/\left(n-nd\right) \end{array} \end{array}$$

Na fórmula, o VC é o valor medido da característica; VSC é o valor medido da subcaracterística; nsc é o número de subcaracterística; m (1) se a resposta for positiva e (0) caso a resposta seja negativa; n é o número total de medidas; nd é o número de questões descartadas. Para transformação dos valores de VC e VSC em índices, seus valores foram multiplicados por 100.

Foram consideradas as respostas concordo totalmente e concordo parcialmente como respostas positivas, e discordo parcialmente ou discordo totalmente, como respostas negativas^(28)^. Foram considerados valores esperados para características e subcaracterísticas, valores acima de 70%, conforme ABNT NBR ISSO/IEC 14598-6^(27)^.

As sugestões dos juízes foram categorizadas em pontos positivos e negativos. Os juízes foram codificados com a letra J e numerados de 1 a 10, a fim de manter o anonimato. Posteriormente, foi realizada a síntese das sugestões para sinalização se seriam acatadas ou não.

## RESULTADOS

Os dez avaliadores do TB*App* eram enfermeiros, dois de cada região do Brasil, sendo nove mulheres, com média de idade de 44,5 anos, variando entre 31 e 70 anos. Seis juízes possuíam entre 10-20 anos de formação e quatro possuíam mais de 20 anos. Quanto à titulação, cinco eram doutores, três eram mestres e dois eram especialistas, com área de especialização predominante em saúde pública. Tomando como referência a origem dos participantes em relação às cinco regiões do Brasil, não houve diferença entre as respostas dos integrantes de cada uma das regiões.

A Tabela 1 mostra a distribuição da avaliação de conteúdo feita pelos juízes especialistas relativas ao objetivo, estrutura, apresentação e relevância. Todos os domínios apresentaram IVC acima de 0,9, ou seja, não exigindo ajuste adicional. O item “O aplicativo está apropriado ao nível sociocultural do público-alvo proposto” do domínio “estrutura” obteve concordância igual a 70% e IVC igual a 0,70, sendo considerado válido, porém, considerando que foi o item que obteve o IVC mais baixo, foram realizados ajustes, conforme sugerido pelos juízes especialistas.

**Tabela 1:** Avaliação de conteúdo do TB*App* por juízes especialistas quanto à objetividade, estrutura e apresentação, relevância, Manaus, Amazonas, Brasil, 2021

Domínios avaliados	Item	DT	DP	CP	CT	IVC	IVC global
Objetivo	É coerente com as necessidades das pessoas adoecidas por TB	0	0	30%	70%	1	0,96
	É coerente do ponto de vista do processo de enfrentamento da TB	0	0	50%	50%	1	
	Promove a mudança de comportamento e atitude	0	10%	60%	30%	0,9	
	Pode ser divulgado no meio científico da área da TB	0	0	0%	100%	1	
	Atende aos objetivos de instituições que trabalham com a TB	0	10%	30%	60%	0,9	
Estrutura e apresentação	O aplicativo é apropriado para pessoas adoecidas por TB	0	0	50%	50%	1	0,91
	As mensagens estão apresentadas de maneira clara e objetiva	0	0	50%	50%	1	
	As informações apresentadas estão cientificamente corretas	0	0	30%	70%	1	
	O aplicativo está apropriado ao nível sociocultural do público-alvo proposto	0	30%	30%	40%	0,7	
	O conteúdo do aplicativo possui sequência lógica	0	10%	10%	80%	0,9	
	As informações estão bem estruturadas em concordância e ortografia	0	10%	60%	30%	0,9	
	O estilo da redação corresponde ao nível de conhecimento do público-alvo	0	10%	50%	40%	0,9	
Relevância	As dúvidas frequentes retratam aspectos-chave que devem ser reforçados	0	0	30%	70%	1	0,92
	O aplicativo permite a aprendizagem sobre a doença	0	10%	40%	50%	0,9	
	O aplicativo propõe à pessoa adoecida por TB adquirir conhecimento para o seu próprio cuidado	0	10%	30%	60%	0,9	
	O aplicativo aborda os assuntos necessários para a pessoa adoecida por TB	0	10%	30%	60%	0,9	
	Está adequado para ser usado por pessoas adoecidas por TB	0	10%	50%	40%	0,9	
	Os recursos disponibilizados no aplicativo são adequados	0	0	40%	60%	0,9	
IVC GERAL: 0,96 (Domínio 1) + 0,91 (Domínio 2) + 0,92 (Domínio 3) / 3 = 0,93							

*DT - discordo totalmente; DP - discordo parcialmente; CP - concordo parcialmente; CT - concordo totalmente; IVC - Índice de Validade de Conteúdo; TB - tuberculose.*

A Tabela 2 mostra a distribuição da avaliação do TB*App* realizada pelos juízes especialistas em relação à tecnologia, incluindo os domínios funcionalidade, confiabilidade, usabilidade e eficiência. Todas as características e subcaracterísticas apresentaram valores iguais ou maiores a 0,80, isto é, em todas as respostas, obteve-se avaliação positiva. O domínio melhor avaliado foi a funcionalidade, e com menores VCS e VC foi o domínio de usabilidade, mas, ainda assim, com VCS e VC satisfatórios, indicando que a versão final do aplicativo foi considerada adequada para uso.

**Tabela 2:** Avaliação de qualidade do *software* TB*App* por juízes quanto à funcionalidade, confiabilidade, usabilidade e eficiência, Manaus, Amazonas, Brasil, 2021

Domínios avaliados	Item	Resposta obtida		Valor final	
		Positiva	Negativa	VCS	VC
Funcionalidade	O aplicativo dispõe das principais funções para auxiliar na adesão ao tratamento da TB	9	1	90%	90%
	O aplicativo é preciso na execução das suas funções	8	2	80%	
	O aplicativo dispõe de segurança de acesso através de senhas	10	0	100%	
Confiabilidade	O aplicativo apresenta mensagens quando ocorrem falhas	9	1	90%	85%
	O aplicativo informa ao usuário a entrada de dados inválidos	8	2	80%	
Usabilidade	É fácil entender o conceito e aplicação do *software*	9	1	90%	83%
	É fácil de aprender a usar o aplicativo	8	2	80%	
	O aplicativo oferece ajuda de forma clara	8	2	80%	
Eficiência	O tempo de execução do *software* é adequado	8	2	80%	90%
	Os recursos disponibilizados no aplicativo são adequados	10	0	100%	

*VCS - valores medidos da subcaracterística; VC - valores medidos da característica.*

No Quadro 1, estão apresentados exemplos de alterações realizadas no TB*App* após sugestões dos avaliadores. A maior parte das sugestões foi relacionada aos domínios estrutura e apresentação e item “O aplicativo está apropriado ao nível sociocultural do público-alvo proposto”, que obteve IVC = 0,70.

**Quadro 1:** Exemplos de mudanças efetuadas nos subitens do aplicativo de acordo com sugestões dos juízes, Manaus, Amazonas, Brasil, 2021

Item	Subitens	
	Versão anterior	Versão final
Quem são os grupos mais susceptíveis a desenvolver tuberculose?	Pessoas vivendo com HIV/AIDS; Pessoas privadas de liberdade; População em situação de rua; Indígenas; Profissionais de saúde; Contatos de pacientes com tuberculose, principalmente crianças e idosos, e pessoas com diabetes.	Pessoas com HIV/AIDS; Detentos; Moradores de rua; Indígenas; Profissionais de saúde; Pessoas que convivem com pacientes com tuberculose, principalmente crianças e idosos, e pessoas com diabetes.
Comecei a tomar a medicação, mas me senti mal. O que devo fazer?	CONTINUE A TOMAR AS MEDICAÇÕES. É importante que você retorne ao serviço imediatamente, para que o profissional de saúde possa avaliar.	Retorne ao serviço de saúde imediatamente, para que um profissional de saúde possa lhe avaliar. Não pare o tratamento.
Eu preciso parar de beber bebidas alcoólicas durante o tratamento da tuberculose?	SIM. Não se podem tomar bebidas alcoólicas, como cachaças, cervejas, whisky, vinhos, e outros, durante o tratamento, pois há riscos de complicações, como, por exemplo, a hepatite. No entanto, existem pacientes que não conseguem parar de beber, neste caso, é recomendado comunicar ao profissional de saúde que está lhe acompanhando e não deixe de tomar as medicações.	É aconselhável que sim. Não se podem tomar bebidas alcoólicas, como cachaças, cervejas, whisky, vinhos, e outros, durante o tratamento, pois há riscos de complicações, como por exemplo: a hepatite. No entanto, se você tiver dificuldade de parar de beber ou deixar de fazer uso de outras drogas, converse com o profissional de saúde.
Por que é importante realizar o exame de HIV?	A tuberculose é uma doença infecciosa que é predominante em pessoas que vivem com HIV, devido à imunidade comprometida destes pacientes, como é também a principal causa de morte nesse grupo. Por isso, é importante que você realize o teste de HIV devido à relação entre as duas doenças.	O HIV é uma doença silenciosa que compromete a defesa do organismo contra outras doenças, como a tuberculose. Quando o paciente apresenta as duas doenças (TB e HIV), a chance de agravamento é maior. Por isso, torna-se importante a descoberta precoce por meio da testagem de HIV
Por que as pessoas que moram comigo têm que ser examinadas?	Porque a família, geralmente são as pessoas que convivem mais com você, portanto, são as que têm maior risco de se infectar e adoecer, pois mantiveram contato antes do diagnóstico e início do tratamento. É importante que todos sejam examinados, mesmo que não tenham os sintomas, pois existe a possibilidade de seus contatos estarem infectados e com risco de desenvolver a doença nos próximos anos.	Porque as pessoas que moram com você (família) por estarem no mesmo ambiente, têm maior risco de se infectar e adoecer, pois antes do diagnóstico e início do tratamento você libera para o meio ambiente as bactérias causadoras da doença. Nesse caso é importante que todos sejam examinados, os que possuem sintomas através do exame de escarro, e os que não apresentem os sintomas da tuberculose através do exame de Prova Tuberculínica (PT) e radiografia de tórax, pois existe a possibilidade de seus contatos estarem infectados e com risco de desenvolver a doença nos próximos anos.
Estou fazendo o tratamento da tuberculose, quais cuidados devo ter em relação a COVID-19? (inserida questão sobre a COVID-19)	Inserido na segunda versão.	É importante manter o tratamento e ter os seguintes cuidados: o uso de máscaras, lavagem das mãos com água e sabão, o uso de álcool em gel, evitar aglomerações, além de verificar como está seu esquema vacinal contra a COVID-19. Em casos de sintomas como febre, tosse seca, cansaço, dor de garganta, coriza, falta de olfato e paladar procurar o serviço.

No Quadro 2, estão apresentados os pontos positivos e negativos do aplicativo de acordo com os juízes especialistas.

**Quadro 2:** Pontos positivos e negativos apontados pelos juízes especialistas, Manaus, Amazonas, Brasil, 2021

Domínios	Pontos positivos	Pontos negativos
1. Objetivos	Auxílio para instituições e profissionais de saúde no enfrentamento da TB e na dificuldade de adesão ao tratamento.	Aceita o registro das tomadas dos medicamentos, mas não garante a observação da tomada.
2. Estrutura e aparência	Textos claros e objetivos.	Respostas muito técnicas considerando o perfil das pessoas atingidas pela TB.
3. Relevância	Indicaria para as pessoas que se tratam ou tratam de pessoas com TB.	Sem destaque.
4. Funcionalidade	O aplicativo é uma excelente ferramenta a ser utilizada durante o tratamento.	Sem destaque.
5. Confiabilidade	Informações corretas.	Sem destaque.
6. Usabilidade	O uso do aplicativo é simples, de fácil manuseio, com informações objetivas e claras.	População mais vulnerável pode não ter acesso ou não ter facilidade em manusear a ferramenta.
7. Eficiência	Bom tempo de resposta e recursos disponibilizados adequados.	Dificuldade para recuperação da senha.

*TB – tuberculose.*

## DISCUSSÃO

Aplicativos móveis criados a partir de pesquisas científicas são relevantes para a implementação na prática, uma vez que tendem a ser analisados e testados por profissionais que conhecem as necessidades do público-alvo^(29, 30)^. O processo de validação de uma tecnologia, quando produzida de forma adequada e validada, poderá contribuir para modificar a realidade dos sujeitos a quem se destina^(31)^.

O TB*App* foi validado em relação a sete domínios, organizados em conteúdo e tecnologia. Os resultados dessa validação permitem afirmar que o TB*App*, do ponto de vista de pesquisadores da área e daqueles que integram a gestão da prática de atenção das pessoas com TB, vinculados ao PCT, tem conteúdo adequado e a tecnologia atende às características necessárias para um aplicativo. A literatura aponta que o processo avaliativo e sugestivo a partir das diferentes visões pelos juízes tende a contribuir para o escopo técnico-científico da tecnologia^(32, 33)^.

Outro aspecto relevante da validação efetuada é que o TB*App* foi avaliado por juízes enfermeiros das cinco regiões do Brasil com experiência na área de saúde pública e TB, e não houve diferença entre suas respostas, indicando que ele poderia ser adequado para uso em todo país.

O reconhecimento da qualidade do aplicativo validado pelos juízes teve como principal sugestão a mudança da linguagem utilizada, uma vez que alguns avaliadores consideraram que nem sempre estava clara para o público-alvo, o que explica a avaliação dada à questão “O aplicativo está apropriado ao nível sociocultural do público-alvo proposto”, que obteve o IVC mais baixo (0,7). As sugestões foram consideradas pertinentes pelos pesquisadores que efetuaram alterações no conteúdo final do TB*App*.

A linguagem também foi apontada como item relevante de ajuste em estudo de validação de aplicativo móvel para acompanhamento do tratamento da infecção latente de TB, cujas mudanças consideraram os relatos dos pacientes relacionados à clareza e simplicidade^(34)^, mostrando a importância de as tecnologias digitais possuírem linguagem clara e acessível, garantindo o entendimento por qualquer pessoa, independentemente do nível sócio-cultura-educacional.

Muitos pontos positivos foram destacados pelos avaliadores em relação à ferramenta, sendo unânimes sobre a divulgação da ferramenta no meio científico. Apesar do crescimento de aplicativos na área da saúde, a grande maioria é criada por desenvolvedores particulares, não está vinculada a projetos de pesquisa e não é divulgada no meio científico^(25)^.

Um dos avaliadores ressaltou que o TB*App* não garante a observação da tomada da medicação. Vale destacar que a tecnologia proposta não visa substituir o TDO, cuja observação da ingesta da medicação deve ser feita por um profissional de saúde ou por uma pessoa treinada, no mínimo, três vezes na semana na fase de ataque e, no mínimo, duas vezes na fase de manutenção, conforme preconização da Organização Mundial da Saúde e diretrizes do Ministério da Saúde^(9)^. A proposta do TB*App* é ser uma ferramenta para o autocuidado, sendo capaz de proporcionar o empoderamento ao usuário durante o seu tratamento, ajudando-o com lembretes de estímulo e com maior informação sobre sua doença e importância da regularidade do tratamento.

Métodos de aconselhamento e lembretes têm sido considerados adequados para pessoas com TB ativa, destacando-se as tecnologias digitais de adesão^(6)^. Além disso, os resultados de revisão sistemática mostraram que há maiores taxas de sucesso do tratamento com o uso de intervenções de adesão, tais como educação, lembretes e tecnologias digitais de saúde, o que reforça a importância do TB*App* desenvolvido, uma vez que ele traz lembretes para a tomada da medicação e mensagens motivadoras, além de conteúdo educativo sobre a doença, exames e tratamento^(35)^.

É de suma importância a criação das tecnologias móveis na área da enfermagem, como a proposta no presente estudo, no entanto essas tecnologias não têm como objetivo substituir a consulta de enfermagem, mas devem atuar de forma complementar às consultas, sendo uma estratégia que possibilite o empoderamento do paciente sobre sua condição de saúde e do seu papel na melhoria de sua qualidade de vida^(36)^.

Tecnologias que inovam a comunicação com as pessoas atendidas na área da saúde estão ampliando o acesso às informações e potencializando a adesão terapêutica^(37)^. O TB*App* foi considerado um aplicativo adequado para uso na APS. No entanto, é importante ponderar as barreiras sociais e econômicas existentes, pois, apesar de a maioria da população brasileira possuir celulares, nem todos têm disponibilidade de *smartphones* ou de outros dispositivos móveis ou mesmo dispositivos com tecnologia suficiente que permita baixar aplicativos^(38)^. Uma alternativa sugerida por pesquisadores foi o fornecimento de *smartphones* às pessoas adoecidas por TB durante o tratamento associado a um treinamento sobre como usar o aplicativo, com possível devolução do aparelho após a conclusão do tratamento com sucesso^(^38-39^)^. Essa sugestão, apesar de interessante, não expressa a realidade dos municípios brasileiros, que estão distantes da possibilidade de terem orçamentos que permitam essas aquisições.

O desenvolvimento do TB*App* está alinhado com os Objetivos de Desenvolvimento Sustentável da Organização das Nações Unidas para 2030, em especial seu Objetivo 3.3: até 2030, acabar com as epidemias de AIDS, TB, malária e doenças tropicais negligenciadas, e combater a hepatite, doenças transmitidas pela água e outras doenças transmissíveis^(40)^.

A contribuição do TB*App* é no sentido de oferecer um dispositivo de fácil acesso às pessoas com TB que os ajudará a controlar melhor sua condição de saúde e disponibilizará aos profissionais de saúde, em especial os enfermeiros, uma ferramenta para obter informações e poder ajudar as pessoas com TB, mesmo que de forma remota, interagindo por meio do aplicativo. Com isso, considera-se que o aplicativo validado pode ser utilizado na APS, contribuindo com a disponibilização de informações pertinentes e de qualidade.

A tecnologia validada difere de outras desenvolvidas no Brasil e une diferentes funcionalidades das tecnologias produzidas em outros países^(16, 17)^. Considera-se o aplicativo desenvolvido, *TBApp,* como uma ferramenta criativa e inovadora para as pessoas adoecidas por TB na APS no município de Manaus. O aplicativo permite o registro diário da tomada da medicação pela pessoa adoecida, além de mensagens-lembretes, com *interface* educativa sobre os aspectos gerais da TB e *interface* para sistema *Web* (TB*Site*) para o uso restrito dos profissionais de saúde e gestores e monitoramento dos casos acompanhados pelo TB*App*, permitindo gerar estatísticas por unidade, distrito e município.

### Limitações do estudo

Como limitação deste estudo, pode-se indicar a não participação de profissionais de saúde que atendem pessoas com TB, o que poderia contribuir para uma análise operacional em relação ao uso da tecnologia. No entanto, é importante ressaltar que essa questão será considerada em estudos futuros, buscando a inclusão desses profissionais para enriquecer a compreensão sobre a implementação e o impacto do aplicativo na prática dos profissionais que atuam diretamente na atenção a pessoa com TB.

### Contribuições para a área da enfermagem

Os resultados da validação do TB*App* para a gestão do autocuidado de pessoas com TB mostram que o mesmo tem o potencial de melhorar o cuidado prestado pelos enfermeiros na APS. O aplicativo em questão representa uma alternativa adicional no cuidado centrado na pessoa com TB, uma vez que sua aplicabilidade foi previamente identificada. A divulgação e a adoção da tecnologia pela população-alvo podem promover o autocuidado, aumentar o conhecimento sobre o tema e trazer melhorias significativas na qualidade de vida.

## CONCLUSÕES

Este estudo avaliou o TB*App* para o autocuidado de pessoas com TB na APS. O aplicativo, segundo avaliação dos juízes, mostrou-se eficaz, funcional, confiável, com boa usabilidade e relevância.

O desenvolvimento dessa proposta tem potencial para impactar na morbimortalidade da doença por meio da promoção ao autocuidado entre as pessoas adoecidas por TB, como também uma ferramenta de acompanhamento de casos, auxiliando os programas de controle no alcance dos indicadores operacionais para eliminação da doença como problema de saúde pública.

A participação de especialistas envolvidos com gestão e a pesquisa na temática da TB potencializaram a qualidade do aplicativo. Espera-se que essa tecnologia possa servir de referência e ser utilizada em outras localidades do Brasil, assim como em países com alta incidência de TB, pela importância das ferramentas digitais em saúde.

Pesquisas posteriores para avaliar a ferramenta com o público-alvo a partir de sua utilização nos serviços poderão identificar o impacto do TB*App* nos indicadores da doença, além de mobilizar o desenvolvimento de novas tecnologias para o controle da TB.

## Data Availability

https://doi.org/10.48331/scielodata.TCLER5
